# Neuroimaging Studies of Suicidal Behavior and Non-suicidal Self-Injury in Psychiatric Patients: A Systematic Review

**DOI:** 10.3389/fpsyt.2018.00500

**Published:** 2018-10-16

**Authors:** Carmen Domínguez-Baleón, Luis F. Gutiérrez-Mondragón, Adrián I. Campos-González, Miguel E. Rentería

**Affiliations:** ^1^Licenciatura en Ciencias Genómicas, Centro de Ciencias Genómicas, Universidad Nacional Autónoma de México, Cuernavaca, Mexico; ^2^Department of Genetics & Computational Biology, QIMR Berghofer Medical Research Institute, Brisbane, QLD, Australia; ^3^Faculty of Medicine, The University of Queensland, Herston, QLD, Australia

**Keywords:** neuroimaging, psychiatric patients, self-harm, suicide attempt, depression, schizophrenia, bipolar disorder

## Abstract

**Background:** With around 800,000 people taking their own lives every year, suicide is a growing health concern. Understanding the factors that underlie suicidality and identifying specific variables associated with increased risk is paramount for increasing our understanding of suicide etiology. Neuroimaging methods that enable the investigation of structural and functional brain markers *in vivo* are a promising tool in suicide research. Although a number of studies in clinical samples have been published to date, evidence about neuroimaging correlates for suicidality remains controversial.

**Objective:** Patients with mental disorders have an increased risk for both suicidal behavior and non-suicidal self-injury. This manuscript aims to present an up-to-date overview of the literature on potential neuroimaging markers associated with SB and NSSI in clinical samples. We sought to identify consistently reported structural changes associated with suicidal symptoms within and across psychiatric disorders.

**Methods:** A systematic literature search across four databases was performed to identify all English-language neuroimaging articles involving patients with at least one psychiatric diagnosis and at least one variable assessing SB or NSSI. We evaluated and screened evidence in these articles against a set of inclusion/exclusion criteria and categorized them by disease, adhering to the PRISMA guidelines.

**Results:** Thirty-three original scientific articles investigating neuroimaging correlates of SB in psychiatric samples were found, but no single article focusing on NSSI alone. Associations between suicidality and regions in frontal and temporal cortex were reported by 15 and 9 studies across four disorders, respectively. Furthermore, differences in hippocampus were reported by four studies across three disorders. However, we found a significant lack of replicability (consistency in size and direction) of results across studies.

**Conclusions:** Our systematic review revealed a lack of neuroimaging studies focusing on NSSI in clinical samples. We highlight several potential sources of bias in published studies, and conclude that future studies should implement more rigorous study designs to minimize bias risk. Despite several studies reporting associations between SB and anatomical differences in the frontal cortex, there was a lack of consistency across them. We conclude that better-powered samples, standardized neuroimaging and analytical protocols are needed to continue advancing knowledge in this field.

## Introduction

Intentional self-harm defies the human intrinsic drive of self-preservation. However, both suicidal behavior (SB) and non-suicidal self-injury (NSSI) are surprisingly common in the population (1). Every year, more than 800,000 people around the world die by suicide (2). In fact, suicide is the second leading cause of death in people aged between 15 and 29 (2), plus it is increasingly recognized as a concerning public health issue in both developed and developing nations alike. Therefore, better prediction, prevention and intervention strategies are urgently needed.

It is estimated that for every completed suicide, there are between 10 and 20 attempts (3). Lifetime prevalence of SB is 9.2% for suicidal ideation, 3.1% for suicidal planning, and 2.7% for suicide attempts (4). Notably, lifetime prevalence of NSSI is estimated between 4 and 6% (including self-cutting, biting, or burning) in adult community samples (5, 6), but it is substantially higher in adolescents (14–47%) (7–9) and clinical samples (21–61%) (5).

The etiology of SB and NSSI is complex and knowledge about their underlying neurobiological mechanisms is limited (10). Several biological pathways have been implicated in the development and progression of NSSI and SB. Specifically, endogenous opioid deficiencies and altered levels of endocannabinoids in the brain have been associated with depression, anxiety, and suicide-related disorders (11, 12). Historically, the study of NSSI and SB has been hindered due to their behavioral nature, and by the common assumption that these behaviors are symptoms or consequences of other underlying mental disorders (13). Hence, despite their preventable nature, it is difficult to detect suicidal tendencies in time to prevent a fatal outcome, as the most common method for risk assessment consists of asking the patient whether he or she has experienced any type of suicidal thoughts. However, given that the topic is considered “taboo” and carries a big stigma in many societies, it is not uncommon for individuals to restrain from communicating their true intentions (14).

SB and NSSI show a strong relationship with mental health disorders (15). In fact, suicide is the most common cause of premature death in patients with major psychotic and mood disorders and up to 90% of individuals who commit suicide present at least one (often undiagnosed or untreated) axis I major psychiatric disorder (16–18). Some psychiatric conditions known for having increased suicide risk include major depressive disorder (MDD), bipolar disorder (BIP), schizophrenia (SCZ), and schizoaffective disorders and borderline personality disorder (BPD), among others (15). A recent meta-review concluded that these conditions are also associated with an increased risk of all-cause mortality and self-harm (15). Although NSSI and SB are routinely assessed as secondary items for some mental disorders, there is considerable variation in how that assessment is made. For instance, the timescale, degree of severity and specific constructs (e.g., ideation, attempt, or intent) vary widely even within single disorders such as MDD (13).

While knowledge about the underlying mechanisms of SB and NSSI remains elusive (19), it is acknowledged that individual genetic factors in combination with environmental stressors influence suicidal outcomes (20). A twin study showed that genetic factors explain a significant part of the variance in NSSI (37% for males and 59% for females) and SB (41 and 55%, respectively) (20). Furthermore, these behaviors are strongly correlated (*r* = 0.49−0.61), and the correlation is largely explained by shared genes (62 and 76% for males and females, respectively) (20). Notably, it is still unknown to what extent the genetic and neural mechanisms that lead to a suicidal attempt are common or distinct across different psychiatric disorders.

Neuroimaging methods, such as magnetic resonance imaging (MRI), allow for the non-invasive interrogation of brain structure and function *in vivo* (21). By comparing groups of patients and healthy controls and applying statistical methods that control for possible confounding covariates such as age, sex, ethnicity, or treatment, it is possible to explore the neural correlates of suicide vulnerability with unprecedented detail. Nonetheless, as with any other scientific approaches, a set of study design principles should be implemented to avoid possible confounding factors and sources of bias to affect study outcomes.

Here we present a systematic review of the literature on neuroimaging studies of SB and NSSI in patients with mental disorders. In addition to summarizing the results of published studies, we assessed potential sources of bias following the PRISMA guidelines. We hypothesized that neuroanatomical differences associated with SB and NSSI would be consistent within disorders. Furthermore, given that affective mental disorders are genetically correlated (22), we also expected some commonalities across disorders to emerge. Overall, we hope to be able to provide a valuable collection of information that can enable more powerful, better-designed analyses of SB and NSSI; also to allow a deeper understanding of these conditions that can ultimately lead to more effective prevention and intervention strategies.

## Materials and methods

### Literature search

**Figure 1:**
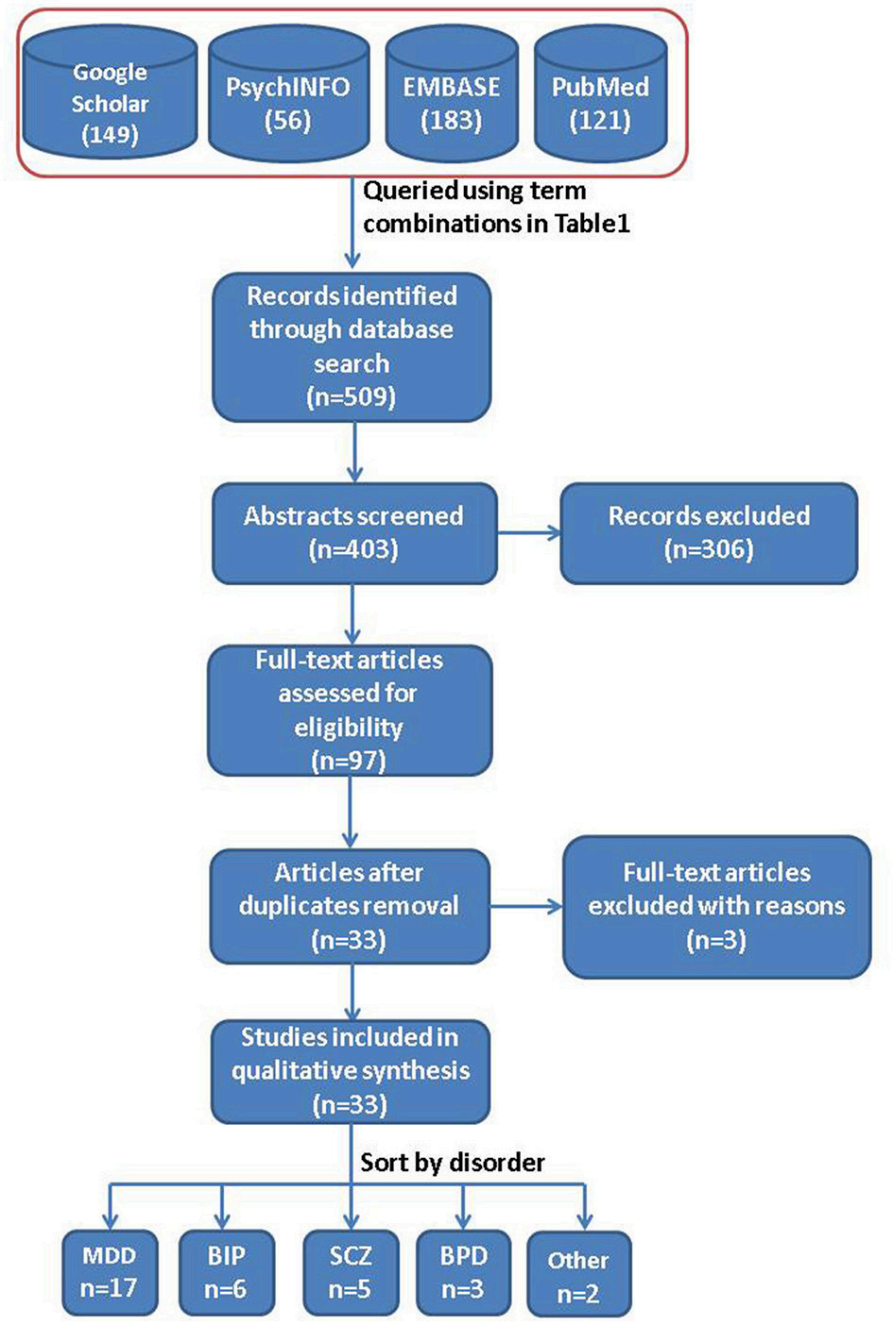
General overview of the methodology. Flow diagram depicting database sources and the steps performed for this systematic review.

Two reviewers conducted literature searches in Google Scholar, PsycINFO, EMBASE, and PubMed for neuroimaging articles investigating SB (ideation, planning, or attempt) and NSSI in patients with psychiatric disorders. We defined three sets of keywords, comprising (i) self-harm and suicide terms, (ii) neuroimaging terms, and (iii) psychiatric disorder terms (Table 1 and Supplementary Material). For each list, we included synonyms or equivalent terms for each of the terms. Subsequently, we used combinations of keywords (one from each category) to systematically query the databases (Figure 1).

**Table 1:** Keywords used to query the bibliographic databases.

**Suicidal behavior/NSSI**	**Neuroimaging**	**Psychiatric disorders**
Self-harm, suicide, suicide attempt, suicidal behavior, suicidal ideation, self-mutilation, self-injury, self-poisoning.	Magnetic-resonance imaging, MRI, brain imaging, neuroimaging.	Psychiatry, psychiatric patients, mental disorder, mental health, psychiatric diagnosis, depression, mdd, major depressive disorder, schizophrenia, bipolar disorder, anorexia nervosa, bulimia nervosa, post-traumatic stress disorder, ptsd, alcohol use disorder, substance use disorder, cannabis, alcoholism, borderline personality disorder, anxiety, alcohol abuse, anxiety disorders, eating disorders.

### Inclusion criteria

In this review, we focused only on structural MRI studies, and not those involving computed tomography or functional MRI techniques. We included only original articles written in English. Therefore, unpublished studies, non-peer-reviewed articles, articles published in a language other than English, case reports, conference abstracts, meta-analyses, review articles, editorials, and articles not assessing neuroimaging phenotypes relative to suicidal symptoms were excluded. The inclusion or exclusion of an article was assessed independently by three of the authors, and in cases of disagreement it was discussed case by case until an agreement was reached.

### Data collection process

All published articles relating suicidality with neuroanatomical phenotypes were thoroughly read by at least three authors. Information on cortical or subcortical phenotype comparisons between patients with suicidal symptoms (as reported by the study) and healthy controls or non-suicidal patients was extracted from tables, figures and the main text of the results section of each article reviewed. Each author contributed to creating and corroborating Tables 2–7. Measurements were collected as standardized values or statistical differences (*Z*-scores, Cohen's d, *F*-score, etc.) in volumetric, thickness, or surface area measurements, and stored as a simplified outcome variable assessing differences between suicidal cases vs. non-suicidal cases and/or healthy controls (Tables 2–7).

**Table 2:** Main characteristics of articles regarding Major Depressive Disorder and Suicide.

**References**	***n***	**Country**	**Gender**	**Age (s.d.)**	**Brain phenotypes**	**MRI**	**Diagnosis**	**Findings**
Gosnell et al. (23)	*n* = 60 (40 patients, 20 controls)	USA	28 F/32 M	MDD suicidal attempt:28.9 (9.98), MDD no suicidal attempt:29.25 (11.1), healthy controls:28.9 (10.0)	Cortical lobes (Frontal, temporal, parietal, occipital), corpus callosum, thalamus, insula, limbic structures, basal ganglia, hippocampus, amygdala.	T1-weighted images on a 3T Siemens Trio MR scanner.	C-SSRS, PHQ, WHOA, DSM-IV	Reduction in right hippocampal volume of the suicidal group compared to healthy controls and frontal and temporal lobe volumes of the suicidal group were diminished compared to the depressed patients.
Colle et al. (24)	*n* = 63 patients	France	~50% F/M	MDD no suicidal attempt: 47.7(12.6), MDD suicidal attempt: 44.2(11.9). Past:47.6(13.4), Acute: 40.2(13.4)	Hippocampal volume.	1.5 or 3-T Philips scanner, SPM5 processing	Hamilton Depression Rating Scale (HDRS) >17, DSM-IVTR.	Depressed patients with a history of suicide attempts had smaller hippocampus than depressed patients without such a history. (TV)
Taylor et al. (25)	*n* = 165 (74 patients, 91 controls)	USA	108 F/57 M	MDD no suicidal: 37.5 (8.9), MDD suicidal: 33.5 (9.1), healthy controls: 29.9 (9.1)	Regional white and gray matter volumes, cortical thickness. Orbitofrontal cortex, cingulate cortex, insula, amygdala, parahippocampus, thalamus and basal ganglia.	Sagittal T1-weighted FreeSurfer processing	DSM-IV, Montgomery–Asberg Depression Rating Scale (MADRS) >15	Depressed group with thoughts of death did exhibit reduced cortical thickness in the left frontal, temporal, parietal regions and the insula but not in regional GMV when compared to the group without thoughts of death.
Peng et al. (26)	*n* = 66 (38 patients, 28 controls)	China	38 F/28 M	MDD suicidal attempt: 27.75(7.21), MDD no suicidal attempt: 31.06 (7.39), healthy controls: 28.61(5.45)	Gray matter volume of the limbic cingulate gyrus, the middle temporal gyrus, the parietal region and the insula.	Philips 3 T, T1-weighted 3D SPM8 processing	Hamilton Depressive Rating Scale (HDRS), DSM-IV, Zung's Self-Rating Depression Scale (SDS).	Patients with a suicide history showed significantly decreased GMV in the right middle temporal gyrus and increased GMV in the right parietal lobe when each group was compared to healthy controls. The suicidal group had a decreased GMV in left limbic cingulate gyrus compared to the non-suicidal group.
Dombrovski et al. (27)	*n* = 52 (33 patients, 19 controls)	USA	30 F/22 M	MDD suicidal attempt: 66.0 (6.4), MDD no suicidal: 67.7 (7.0), healthy controls: 70.5 (7.5)	Basal ganglia gray matter integrity.	T1-weighted images, automated labeling pathway (ALP) processing	SCID/DSM-IV, HDRS-17	Suicide Attempters had lower putamen gray matter voxel counts and lower voxel counts in associative and ventral striatum.
Wagner et al. (28)	*n* = 60 (30 patients, 30 controls)	Germany	50 F/10 M	MDD high-risk for suicide: 41.0 (12.5), MDD low-Risk for suicide: 34.1 (10.5), healthy controls: 35.1(10.4)	Cortical thickness, dorsolateral prefrontal cortex (DLPFC), ventrolateral prefrontal cortex (VLPFC)	1.5T Siemens scanner, T1-weighted images, FreeSurfer processing	DSM-IV diagnosis, 18 on the 21-item HDRS	Patients with depression and a high risk for suicide had a substantially thinner cortex in the left DLPFC, VLPFC and the anterior cingulate when compared against non-high risk patients.
Cyprien et al. (29)	*n* = 435 (201 patients, 234 controls)	France	222 F/213 M	MDD suicidal attempt: 72.2 (4.3), MDD no suicidal attempt: 71 (3.8), healthy controls: 71 (3.8)	Corpus Callosum volume	T1-weighted imaging, Analyze 9.0 processing	MINI/DSM-IV criteria, Center for Epidemiological Studies-Depression scale (CES-D)	The area of the posterior third of corpus callosum was significantly smaller in suicide attempters than in affective controls and healthy individuals.
Wagner et al. (30)	*n* = 60 (30 patients, 30 controls)	Germany	50 F/10 M	MDD High-risk: 41.0 (12.5), MDD Low-risk: 34.1(10.5), Controls: 35.1 (10.4)	Regional Gray Matter density	Sagittal T1-weighted SPM2 processing	DSM-IV, 18 on the 21-item HDRS	Depressed individuals with higher risk for suicide presented diminished gray matter density in a fronto-striato-limbic network in comparison to healthy controls and in caudate and rostral anterior cingulate cortex when compared to non-high risk patients.
Hwang et al. (31)	*n* = 96 (70 patients, 26 controls)	Taiwan	All Male	MDD suicidal attempt: 79.1(5.6), MDD no suicidal attempt: 79.6 (5.1), healthy controls: 79.5 (4.3)	Regional volumes. GM (insula, posterior cingulate) WM (subcallosal cingulate cortex, floor of lateral ventricles, parahippocampal region, insula, cerebellum).	Siamens 2T T1-weighted imaging, SPM2 processing	17 on the 21-item Hamilton Depression Rating Scale (HDRS), DSM-IV	Suicidal depression was associated with decreased gray matter and white matter volume in the frontal, parietal and temporal lobes, and the insula, lentiform nucleus, midbrain, and cerebellum when compared with non-suicidal individuals.
Pompili et al. (32)	*n* = 99 (all patients)	Italy	57 F/42 M	No suicidal attempt:47.27 (14.54), Suicidal attempt: 45.57 (16.10)	Total White Matter Hyperintensities	T1 & T2-weighted images	DSM-IV-TR diagnosis of major affective disorders (MDD, BIP type I, BIP type II, subst. Abuse), MINI/DSM-IV-TR.	The presence of periventricular white matter hyperintensities was robustly associated with **suicidal behaviors**.
Monkul et al. (33)	*n* = 34 total (17 patients, 17 controls)	USA	All Female	Suicidal: 31.4 (13.9), Non Suicidal: 36.5 (7.5), Healthy controls: 31.3 (8.3)	Regional GM and WM volumes (orbitofrontal cortex, cingulate, amygdala and hippocampus)	1.5T GE Signa Imaging. Signa 5.4.3 software	Structured Clinical Interview for DSM-IV (SCID). (HDRS).	Suicidal depressed patients showed a smaller GMV in the right and left orbitofrontal cortex compared with healthy subjects. Suicidal patients presented larger right amygdala volumes when compared to non-suicidal patients.
Pompili et al. (32)	*n* = 65 (all patients)	Italy	41 F/24 M	MDD suicidal attempt: 42.17(13.51) MDD no suicidal attempt: 44.61 (13.95)	White Matter Hyperintensities	Axial and coronal T2-weighted Axial and Sagittal T1-weighted	MDD, BIP MINI/DSM-IV diagnosis	The prevalence of white matter hyperintensities was significantly higher in subjects with **past suicide attempts**.
Ehrlich et al. (34)	*n* = 102 (all patients)	USA	68 F/34 M	26.7(5.5)	White matter hyperintensities	T2-weighted	DSM-IV diagnosis.	The prevalence of periventricular white matter hyperintensities was significantly higher in subjects with **past suicide attempts**
Ehrlich et al. (35)	*n* = 153 (all patients)	USA	41 F/112 M	Psychosis: 16.0(3.3), BIP: 14.9 (3.5), Depression: 15.1(2.8)	White matter hyperintensities	T2-weighted	DSM-IV diagnosis	White matter hyperintensities were significantly associated with a higher prevalence of **past suicide attempts**
Ahearn et al. (36)	*n* = 40 (all patients)	USA	34 F/6 M	MDD suicidal attempt: 66.0 (5.8), MDD no suicide attempt: 66.4 (5.7)	Gray matter hyperintensities	1.5 T and T2 weighted imaging	The Hamilton Depression Scale (HDRS)	Unipolar patients with a history of a **suicide attempt** demonstrated significantly more subcortical gray matter hyperintensities compared with patients without such a history
Lee et al. (37)	*n* = 38 (all patients)	South Korea	20 F/18 M	MDD suicidal attempt: 41.95 (10.81), MDD no suicidal attempt: 41.11 (15.15)	Regional gray matter volume differences.	T1-weighted imaging, SPM8 processing	DSM-IV, HDRS	Compared with suicide non-attempters, **suicide attempters** exhibited decreased GM volume in the left angular gyrus and right cerebellum.
Sachs-Ericsson et al. (38)	*n* = 246 (all patients)	USA	190F/55M	MDD suicidal attempt: 66.74 (6.6), MDD no suicidal attempt: 69.8 (7.5)	White matter lesions (WML)	1.5 T imaging MrX processing	MontgomeryeAsberg Depression Rating Scale (MADRS), DSM-IV, MMSE, DIS.	Higher baseline WML in left hemisphere. **Attempt history** predicted growth in WML.

**Table 3:** Main characteristics of articles regarding Bipolar Disorder and Suicide.

**References**	***n***	**Country**	**Gender**	**Age (s.d.)**	**Brain phenotypes**	**MRI**	**Diagnosis**	**Findings**
Johnston et al. (39)	*n* = 113 (68 patients, 45 controls)	USA	69 F/44 M	BIP suicidal attempt: 20.5 (3.0), BIP no suicidal attempt: 20.5 (3.0), healthy controls: 20.8 (3.3)	Regional Gray matter volumes	Sagittal, T1-weighted, SPM5 processing	DSM-IV criteria	**Attempters** demonstrated significantly lower GMV than non-attempters in the right orbitofrontal cortex and hippocampus, as well as bilaterally in the cerebellum extending into the vermis.
Lijffijt et al. (40)	*n* = 93 (51 patients, 42 controls)	USA	All Female	BIP suicidal attempt: 36.6 (10.7), BIP no suicidal attempt:41.1 (11.3)	Prefrontal cortex gray matter volume	High resolution 3-D T1-weighted, 1.5-Tesla Intera scanner	DSM-IV(SCID, HDRS, (Young Mania Rating Scale) YMRS.	PFCGM (prefrontal cortex gray matter) volume was lower in patients with than without **attempt history** in those with past psychiatric hospitalization.
Nery-Fernandes et al. (41)	*n* = 62 (40 patients, 22 controls)	Brazilian	41 F/21 M	BIP suicidal: 39.8(11.4), BIP non-suicidal: 42.0(8.6), Healthy controls: 37.7(13.5)	Corpus callosum sub-region area: (rostrum, genu, rostral body, anterior midbody, posterior midbody,isthmus, and splenium).	1.5T Sigma Imaging, ANALYZE AVW processing	DSM-IV axis I (SCID-I), HDRS, YMRS.	No differences were observed for any subregion between Bipolar-Suicidal and Bipolar-Non Suicidal groups. There was a significant reduction in the genu and isthmus areas in bipolar patients compared with HC.
Baldaçara et al. (42)	*n* = 62 (40 patients, 22 controls)	Brazilian	41 F/21 M	BIP suicidal attempt:39.94 (11.12), BIP no suicidal attempt: 41.9 (8.9), Healthy controls:37.72 (13.63)	Cerebellar volume	Sagittal T1, 1.5T Symphony Master/Class Siemens	SCID-I, HDRS, YMRS.	No volumetric differences were found between the BIP subjects with **suicidal attempt** and those without such history.
Matsuo et al. (43)	*n* = 47 (20 patients, 27 controls)	USA	All Female	BIP suicidal: 36.2 (10.1), BIP non suicidal: 44.2 (12.5), Healthy controls: 36.9 (13.8)	Corpus Callosum genu, anterior body, posterior body, isthmus and splenium areas	Philips 1.5 T, Axial 3D T1 weighted	DSM-IV/SCID, HDRS, YMRS.	No significant differences among the three groups on any regional CC areas, although the suicidal BIP patients had the smallest areas.
Duarte et al. (44)	*n* = 59 (39 patients, 20 controls)	Brazillian	34 F/25 M	BIP suicidal attempt: 41.1(12.64), BIP no suicidal attempt: 42.26 (11.70), Healthy controls: 37.40 (10.20)	Whole brain exploratory. Hypothesis driven: OFC (superior lateral, middle lateral, inferior lateral and medial); dorsal lateral PFC (DLPFC) (inferior, middle and superior frontal gyri); ACC; amygdala; hippocampus; insula; and thalamus.	Philips 1.5 T 3D T1 weighted	DSM-IV-TR, MINI-PLUS, HDRS	Attempters showed an increase in GMV in the rostral anterior cingulate cortex, insula and orbitofrontal cortex when compared to non-attempters.

**Table 4:** Main characteristics of articles regarding Schizophrenia and Suicide.

**References**	***n***	**Country**	**Gender**	**Age (s.d.)**	**Brain phenotypes**	**MRI**	**Diagnosis**	**Findings**
Besteher et al. (45)	*n* = 87 (37 patients, 50 controls)	Germany	46 F/41 M	SCZ suicidal attempts: 34.4 (12.1), SCZ no suicidal attempts:28.8 (9.7), Healthy controls:29.5 (7.9).	Cortical thickness (CT)	T1-weighted imaging on 1.5-T Siemens. FreeSurfer processing.	DSM-IV	Suicide attempters had cortical thinning in the bilateral caudal middle frontal gyrus, lateral orbitofrontal and superior frontal gyrus, left pars orbitalis and right caudal ACC, pars opercularis and triangularis when compared with healthy controls.
Giakoumatos (46)	*n* = 751 (489 patients, 262 controls)	USA	387 F/364 M	SCZ no suicidal attempt: 35.9(13.3). SCZ suicidal attempt lethality; High:35.6(11.7), Low:36.9(12.2), Healthy controls:38.1(12.5)	Regional Gray matter volume in temporal, parietal, frontal regions.	High-resolution isotropic T1-weighted imaging, FreeSurfer analysis	DSM-IV-TR/SCID	Attempters had significantly less GMV in bilateral inferior temporal and superior temporal cortices, left superior parietal, thalamus and supramarginal regions, right insula, superior frontal and rostral middle frontal regions when compared to non-attempters.
Spoletini et al. (47)	*n* = 100 (50 patients, 50 controls)	Italy	45 F/55 M	SCZ suicidal attempt: 42.9 (11.3), SCZ no suicidal attempt: 39.8 (11.4), Healthy controls: 40.0 (16.6)	Volumetric data of lateral ventricles, thalamus, hippocampus, amygdala, caudate, putamen, pallidum and accumbens	3D T1-weighted, T2-weighted FSL 4.1 software for processing	DSM-IV-TR, MMSE	An increased volume in the right amygdala was observed in **lifetime suicide attempters** compared non attempter and HC. Increased right amygdala volume was correlated to augmented self-aggression.
Aguilar et al. (48)	*n* = 37 (all patients)	Spain	All Male	SCZ suicidal: 37.12 (10.02), SCZ non Suicidal: 42.65 (10.19)	Gray Matter density	1.5 T, 3D T1-weighted, SPM5 processing	DSM-IV	Reduction in gray matter in the left superior temporal lobe and left orbitofrontal cortex was observed in patients with a **record of suicide attempts** when comparing to non-suicidal patients.
Rüsch et al. (49)	*n* = 110 (55 patients, 55 controls)	Italy	42 F/68 M	SCZ suicidal attempt: 30.3 (6.5), SCZ no suicidal attempt: 37.3 (11.6), Healthy controls: 36.0	Inferior frontal region white matter volumes	T1-weighted 3D SPM2 processing	DSM-IV/SCID-I	Increased bilateral inferior frontal white matter volumes were shown by patients **with a previous suicide attempt** as compared with those patients without such history. Among patients, current self-aggression was positively correlated with white matter volume in the aforementioned regions.

**Table 5:** Main characteristics of articles regarding Borderline Personality Disorder and Suicide.

**References**	***n***	**Country**	**Gender**	**Age (s.d.)**	**Brain phenotypes**	**MRI**	**Diagnosis**	**Findings**
Soloff et al. (50)	*n* = 51 (all patients)	USA	41 F/10 M	BPD High lethality attempers: 36.1 (9.2), BPD Low lethality attempers: 27.4 (5.9)	Regional GM volumes mid-inf. orbital frontal cortex, anterior cingulate cortex, middle-superior temporal cortex, insula, hippocampus, parahippocampus, fusiform gyrus, lingual gyrus and amygdala	1.5T Signa Imaging, Sagittal T1-weighted, (DARTEL) in SPM8 processing	DSMIII-R or DSM-IV (SCID), Diagnostic Interview for Borderlines (DIB).	High lethality attempters had diminished GMV in right midsupperior temporal gyrus, right middle inferior orbitofrontal gyrus, right insular cortex, left fusiform gyrus, left lingual gyrus and right parahippocampal gyrus compared to low lethality attempters.
Soloff et al. (51)	*n* = 120 (68 patients, 52 controls)	USA	76 F/44 M	BPD: 28.3 (7.5), Healthy controls: 25.9 (7.2)	Regional GM concentrations mid-inf. orbitofrontal cortex, mid-sup temporal cortex, anterior cingulate, insula, hippocampus, amygdala, fusiform, lingual and parahippocampal gyri	1.5T Sign, Sagittal T1-weightes imaging, SPM5 processing	DSMIII-R or DSM-IV (SCID), DIB.	BPD attempters had diminished gray matter volumes in the insula, the left hemisphere, the middle superior temporal cortex, the right hemisphere, hippocampus, fusiform gyrus, parahippocampus, anterior cingulate and amigdala when compared to healthy controls.
Goodman et al. (52)	*n* = 26 (13 patients, 13 controls)	USA	20 F/6 M	BPD: 15.8 (1.1), Healthy controls: 16.2 (0.8)	Gray and white matter volume in the prefrontal cortex	T1-weighted MP-RAGE	DSM-IV, DIB, BDI.	Number of suicide attempts was positively associated with smaller overall Brodmann area 24 (ACC) volume.

**Table 6:** Main characteristics of articles regarding other affective disorders and suicide.

**References**	***n***	**Country**	**Gender**	**Age (s.d.)**	**Brain phenotypes**	**MRI**	**Diagnosis**	**Findings**
Kim et al. (53)	*n* = 36 (all patients)	Korea	23 F/13 M	Panic Disorder suicidal attempt: 33.42 (14.09), Panic Disorder no suicidal attempt: 34.00 (9.38)	Gray Matter and White Matter volumes	3D T1-FSPGR imaging. SPM5 processing	DSM-IV/SCID.	The VBM analysis revealed no significant intergroup difference in the GM and WM volumes.
Thomas et al. (54)	*n* = 182 (61 patients, 121 controls)	USA	93 F/89 M	PTSD: 11.74 (2.4), Healthy Controls: 11.74 (2.7)	Pituitary volumes	T2-weighted images (GE 1.5- Tesla Unit)	DSM-IV	PTSD subjects with a history of suicidal ideation had larger pituitary volumes than healthy controls.

**Table 7:** Cortical phenotypes associated with SB across disorders.

**Disorder/Area**	**Frontal**	**Prefrontal**	**Orbitofrontal**	**Parietal**	**Temporal**	**Occipital**	**Limbic (Cingulate)**	**Insular**
MDD Wagner et al. (28)		Reduction DLPFC and VLPFC CT					Reduction anterior CT	
MDD Peng et al. (26)				Increase right GM			Reduction left limbic GM	
MDD Wagner et al. (30)	Reduction GM						Reduction anterior, rostral and dorsal GM	
MDD Taylor et al. (25)	Reduction CT			Reduction left CT	Reduction CT			Reduction left CT
MDD Gosnell et al. (23)	Reduction CV				Reduction CV			
MDD Hwang et al. (31)	Reduction GM, WM			Reduction GM, WM	Reduction GM			Reduction GM
MDD Monkul et al. (33)			Reduction GM					
MDD Duarte et al. (44)			Increase GM				Increase GM	Increase GM
BD Johnston et al. (39)			Reduction GM					
BD Lijffijt et al. (40)		Reduction GM						
BP-P Giakoumatos et al. (46)					Reduction fusiform gyri bilaterian inferior and superior GM			
SCZ Besteher et al. (45)	Reduction CT	Reduction CT	Reduction CT	Reduction left superior CT	Reduction CT	Reduction CT	Reduction right caudal anterior CT	Reduction CT
SCZ Giakoumatos et al. (46)	Reduction GM			Reduction GM	Reduction inferior GM	Reduction right cuneus, pericalcarine GM		Reduction right GM
SCZ Aguilar et al. (48)			Reduction GM		Reduction left superior GM			
SCZ Rusch et al. (49)		Increase WM	Increase WM					
BPD Soloff et al. (51)			Reduction bilateral middle inferior GM		Reduction fusiform gyrus, bilateral middle superior GM	Reduction lingual gyrus GM	Reduction left anterior GM	Reduction right GM
BPD Soloff et al. (50)			Reduction right middle inferior GM		Reduction fusiform gyrus, right middle superior gyrus GM	Reduction left lingual gyrus GM		Reduction right and left GM
BDP Goodman et al. (52)							Reduction CV	

*CT, Cortical thickness; CV, Cortical volume; GM, Gray matter volume; WM, White matter volume*.

### Bias risk assessment

Typical bias risk assessment instruments (e.g., Cochrane) are intended for interventional studies such as randomized controlled trials. For case-control studies searching for neurobiological associations, there is, to the best of our knowledge, no gold standard instrument for performing bias risk assessment. Nonetheless, we acknowledge the importance of finding possible sources of bias in the reviewed literature. Thus, we devised a table including possible sources of confounding or bias in structural neuroimaging association studies, including key design, and statistical aspects such as matching suitable controls and cases, assessing absence of mental disorders in the control group, assessing MRI scans for artifacts and pathological findings, etc. Three authors independently filled this table indicating whether a study had a low, medium or high risk of bias for all constructs. As an example, for the construct “controls matched to subjects,” a study that perfectly matched demographics between controls and cases would receive a score of low risk, while a study with only partially matching controls or non-matching controls would receive a score of medium or high risk, respectively. For detailed information on each construct, their interpretation and coding please refer to the Supplementary Material.

## Results

**Figure 2:**
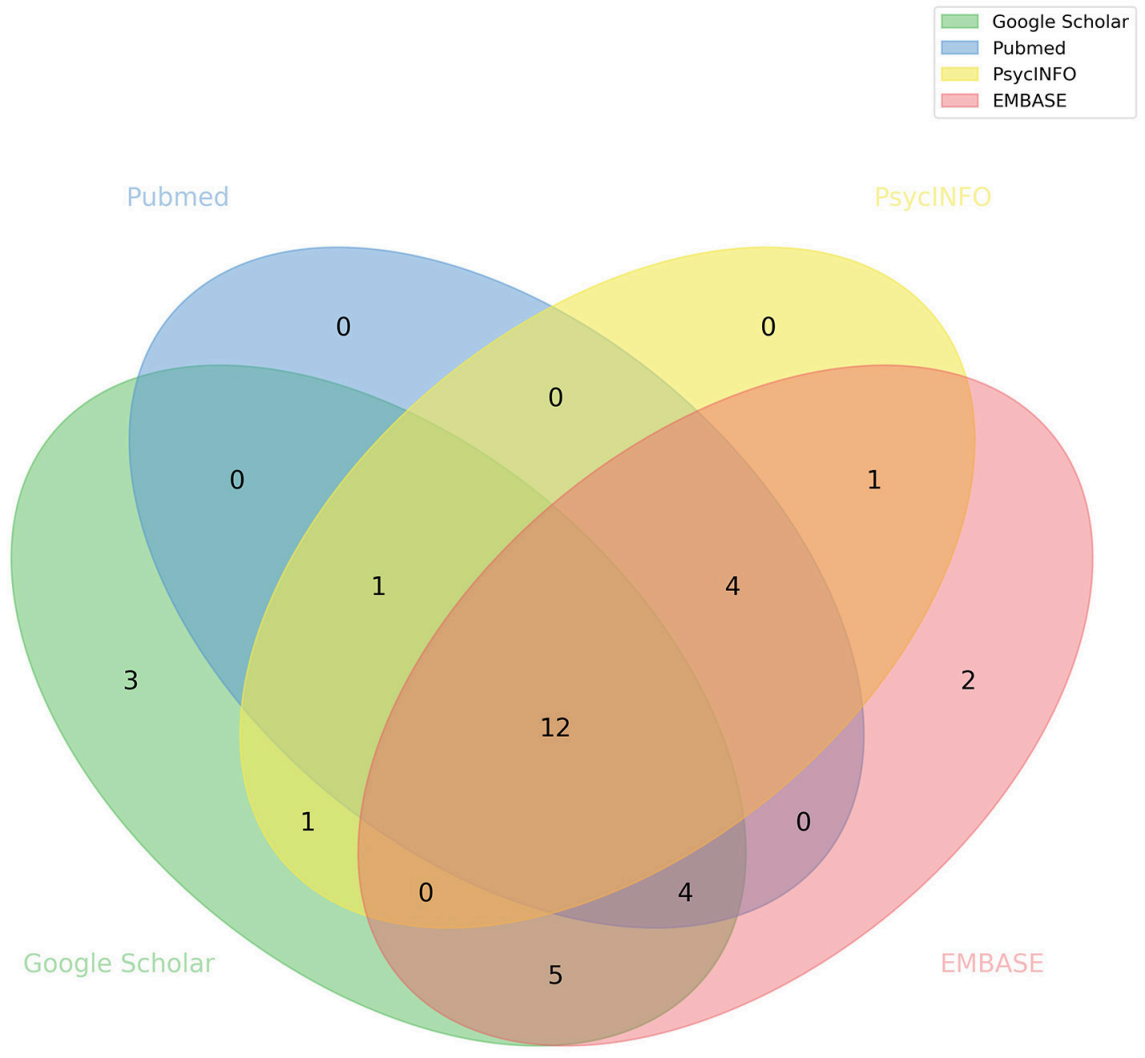
Database source of articles included in this systematic review. Venn diagram illustrating the overlap between studies obtained from different databases.

After applying our quality control and exclusion criteria, we were left with 33 articles (Figure 2). Two meta-analysis studies (21, 55), and a study not assessing imaging phenotypes with SB (56) were excluded. Of the selected studies, the majority were related to MDD (17 out of 33) and mainly focused on reporting gray matter volume differences. Characteristics of each individual study are detailed in Tables 2–5 No single article focusing on NSSI alone was found. Below we summarize the main findings related to specific cortical and subcortical brain regions in MDD, BIP, SCZ, or BPD.

### Major depressive disorder

MDD affects nearl2y 32 million people worldwide (57). The Diagnostic and Statistical Manual of Mental Disorders 5th edition (DSM-5), describes this psychiatric disorder as characterized by depressed mood and/or diminished interest or pleasure, vegetative symptoms such as disturbed sleep or appetite and persistent thoughts of death, suicidal ideation or previous suicide attempts (58). In fact, up to 50% of all completed suicides annually occur within a depressive episode and MDD patients are 20-fold more likely to die by suicide than healthy individuals (59).

Seventeen studies focused on identifying brain regions associated with SB in patients with MDD (the characteristics of each study are summarized in Table 2). Sample size across studies ranged from 34 to 246 individuals aged between 20 and 80 years. Cohorts included participants from the USA, Germany, France, Netherlands and China. With the exception of two studies, all included a combination of both male and female individuals. Most of them compared structural MRI brain phenotypes between depressed suicidal, depressed non-suicidal, and healthy control groups. In addition, the majority of MDD diagnoses were made according to DSM-IV criteria. Phenotypes of interest were mostly gray and white matter regional volumes, but five studies focused on differences in white matter hyperintensities. In this section, we summarize the main findings recovered from these articles, divided into cortical and subcortical phenotypes.

#### Cortical regions

We identified four independent studies suggesting a potential link between the **insular cortex** region and suicidal symptoms in MDD. Taylor et al. (25) described reduced cortical thickness in the left insula of MDD patients with death thoughts, compared to MDD patients without death thoughts, but found no significant difference between healthy controls and any of the depressed groups, as reported in two other studies (26, 28). In another study, Hwang et al. (31) found reduced gray matter volumes within the left and right insula of suicidal MDD patients, compared to non-suicidal MDD patients. Taylor et al. (25) argue that the insula may be relevant because it is a component of the salience network and may potentiate the neural response to negative stimuli through its connections with the amygdala and the cingulate cortex. Moreover, Hwang et al. (31) add that the insula is preferentially engaged in internally generated emotions and functions as a relay signal station to maintain homeostasis.

The **frontal lobe** was consistently reported as potentially implicated in SB within MDD. Taylor et al. (25) found reduced cortical thickness in the left frontal lobe of MDD patients with death thoughts, compared with non-suicidal MDD patients. Hwang et al. (31) and Gosnell et al. (23) reported significantly decreased volumes in the **right, left and total frontal lobe**, both studies made the observations when comparing suicidal vs. non-suicidal MDD patients. Wagner et al. (30) observed differences in regional gray matter density between MDD patients with high risk for suicide and healthy controls in the **right inferior frontal gyrus**. As mentioned by Gosnell et al. (23), the frontal lobe may inhibit the emotional limbic system, which is probably dysregulated in emotionally unstable individuals and thus could cause the characteristic impulsiveness of SB.

Studies also highlighted differences in the **temporal lobe**; Taylor et al. (25) found reduced cortical thickness in MDD patients with death thoughts, compared with MDD patients without death thoughts. Gosnell et al. (23) observed significantly lower right and total temporal lobe volumes, and Hwang et al. (31) reported reduced gray matter volumes within temporal regions when comparing MDD suicidal patients against non-suicidal MDD patients. Also, Peng et al. (26) reported smaller gray matter volumes in the right middle temporal gyrus of MDD suicidal patients compared to healthy individuals. Both Gosnell et al. (23) and Hwang et al. (31) hypothesize that the temporal cortex may be implicated in emotional dysregulation in suicidal individuals.

Three independent studies associated the **cingulate cortex** with SB in MDD. Wagner et al. (28) observed a cortical thickness reduction in the **anterior cingulate** of MDD patients at high risk for suicide, compared to non-high-risk patients. Moreover, Peng et al. (26) found significantly decreased gray matter volume in the **left limbic cingulate gyrus** of suicidal MDD patients with respect to the non-suicidal depressed group. Finally, Wagner et al. (30) found differences in regional gray matter density between MDD patients at high risk for suicide and healthy controls in the **rostral and dorsal part of the anterior cingulate cortex**, a result later replicated by the same group in 2012 (28). As the cingulate is part of the limbic system, involved in emotional formation and processing, it has been proposed to directly influence the SB of depressed patients (26).

Differences in the **parietal lobe** have also been reported. Taylor et al. (25) described reduced cortical thickness in the **left parietal lobe** in MDD patients with death thoughts, compared to MDD patients without death thoughts. Hwang et al. (31) found reduced gray matter and white matter volumes within parietal regions in suicidal MDD patients compared to non-suicidal MDD patients. On the other hand, Peng et al. (26) observed an increase in gray matter volumes in the **right parietal lobe** in suicidal MDD patients compared to healthy individuals. Peng et al. (26) argue that the parietal lobe is connected to other parietal occipital-temporal areas, which have also been suggested to be altered in MDD patients with suicidal symptoms.

In addition, single studies have implicated **other regions** with suicidal symptoms, but these were not replicated in other studies. For instance, Monkul et al. (33) reported gray matter volume reductions in the **orbitofrontal cortex** of MDD suicidal patients when compared to non-suicidal patients, and Wagner et al. (28) found reduced cortical thickness in the **left dorsolateral and ventrolateral prefrontal cortex** when comparing patients at high suicide risk against non-high-risk patients.

#### Subcortical regions

Both Gosnell et al. (23) and Colle et al. (24) found significantly reduced right hippocampal volumes in the suicidal MDD group compared to the healthy controls and non-suicidal MDD patients. This association is consistent with the role of the hippocampus in regulating emotional responses (60) and with findings of hippocampal volumetric decreases as predictors of slower depression recovery (61).

Ahearn et al. (36) reported that MDD patients with a history of a suicide attempt had substantially more subcortical gray matter hyperintensities compared to patients without such a history. Monkul et al. (33) observed larger **right amygdala** volumes in suicidal vs. non-suicidal MDD patients, and Wagner et al. (30) reported a decrease in regional gray matter density between depressed patients at high suicide risk and healthy controls in the **right amygdala–hippocampus formation**. Also, Wagner et al. (30) found differences in regional gray matter density between depressed patients at high risk for suicide and healthy controls in the **caudate nucleus**. Hwang et al. (31) found reduced gray matter volumes within the **lentiform nucleus** when comparing suicidal vs. non-suicidal MDD patients, and reduced gray matter volumes within the cerebellum, an observation later replicated by Lee et al. (37). A cortical volume decrease was reported in single studies in the **corpus callosum** (29) and **putamen** (27) regions.

In the largest neuroimaging study of suicidality in MDD published to date, Rentería et al. (21) conducted a series of meta-analyses in 3,097 individuals, including 1,996 healthy controls, 451 MDD patients with suicidal symptoms, and 650 MDD patients without suicidal symptoms. The meta-analyses did not replicate any previously reported associations between subcortical volume and suicidal symptoms (ideation or planning/attempt) in MDD patients. The study reported only a smaller intracranial volume in MDD patients with suicidal symptoms compared with healthy controls, and a study-wide non-significant trend of smaller subcortical volumes and larger ventricular volumes in suicidal patients compared with healthy controls. Importantly, the authors noted that even with those sample sizes the study was underpowered, thus raising the question of statistical power of previous studies with substantially smaller sample sizes (see section Discussion).

#### White matter hyperintensities

Several studies reported on white matter hyperintensities (WMH) and periventricular white matter hyperintensities (PVH) and their association with past suicide attempts (32, 34, 35). Pompili et al. (32) found that lifetime suicidal ideation in the presence of a history of suicide attempt was positively associated with the presence of WMH, and later (62) showed that suicide attempters were more likely to have higher PVH than non-attempters. The same studies reported that suicidal ideation without a history of suicide attempt was not significantly associated with any measure of WMH, PVH, Deep white matter hyperintensities (DWMH), or subcortical gray matter hyperintensities (32, 34), and that DWMH was not significantly associated with suicidality (35, 62). Ehrlich et al. (34) proposed that MDD patients with PVH may be at higher risk of suicide due to the possible disruption of neuroanatomical pathways, as they are associated with ependymal loss and varying degrees of myelination. Although white matter lesions have been linked to aging, they were found to be associated with suicide attempters in an older cohort (38), further supporting the observed correlation with SB.

### Bipolar disorder

The symptoms of BIP include alterations in mood and energy levels and in the person's ability to execute everyday tasks. The characteristic mood episodes go from manic extremely energetic periods to sad, hopeless depressive episodes (63). Suicide rates among BIP patients are 20-fold or higher than in the general population (64). Our literature search identified six scientific articles that investigated neuroanatomical differences in BIP patients with suicidal symptoms. The main characteristics of the studies are summarized in Table 3. Overall, sample sizes ranged from 47 to 113 individuals; participants in 2 studies were exclusively female, whereas 2 other studies had a combination of males and females; national origin of participants was the USA and Brazil. The articles mainly studied regional brain volumes using structural MRI and followed BIP diagnosis criteria in the DSM-IV.

#### Cortical regions

Given the relatively few neuroimaging studies analyzing suicidality in BIP patients, most putative brain regions (i.e., cortical and subcortical) have been implicated by only a single study. Johnston et al. (39) showed that BIP patients who attempted suicide had a lower gray matter volume in the **orbitofrontal cortex** than non-attempters. In contrast, Duarte et al. have recently reported an increase in GMV in this region and the **limbic** and **insular** lobes (44). Moreover, Lijffijt et al. (40) reported changes in the **prefrontal cortex** while studying BIP with and without a record of previous psychiatric hospitalization; they also found larger gray matter volume in non-hospitalized patients who had made a suicide attempt relative to patients without history, but lower gray matter volume in BIP suicide attempters compared to those without attempts in the previously-hospitalized group. This last observation could indicate that the severity of the disease (which is correlated with the hospitalization record) could explain the prefrontal cortex differences between BIP suicide attempters and non-attempters.

#### Subcortical regions

Contrasting results were found for the cerebellum. Johnston et al. (39) reported significantly lower bilateral gray matter volume in the **cerebellum**, extending into the vermis in the attempter group, compared to non-attempters. On the contrary, Baldaçara et al. (42) had previously reported no total volumetric differences in the **left cerebellum, right cerebellum**, nor the **vermis** between BIP suicide attempters and non-attempters. Importantly, these observations highlight the need for more studies regarding this topic, as more evidence would help clarify this issue.

Johnston et al. (39) reported lower gray matter **hippocampal** volume in BIP suicide attempters compared to non-attempters and healthy controls. Finally, Nery-Fernandes et al. (41) and Matsuo et al. (43) found no significant differences in **total brain**, white matter and gray matter volumes among the BIP suicidal and BIP non-suicidal groups.

### Schizophrenia

SCZ is a chronic, severe and disabling psychiatric disorder that is characterized by the presence of hallucinations, delusions, dysfunctional ways of thinking and agitated body movements (65). Up to 40% of premature mortality related to SCZ can be attributed to suicide (66). Various efforts have been undertaken to identify suicide risk factors in the context of this condition (67, 68), but only a few have investigated neuroimaging correlates. We found five articles (summarized in Table 4), with samples sizes ranging from 37 to 751 individuals. All except one included both male and female participants. Psychiatric diagnoses were conducted according to DSM-IV criteria and cross-group comparisons included schizophrenic suicide attempters, non-attempters and healthy controls.

#### Cortical regions

Four (of five) independent studies found differences in the **frontal lobe** between suicidal SCZ patients and either non-suicidal individuals or healthy controls (HC). Besteher et al. (45) observed a pronounced cortical thinning in the **right DLPFC** (dorsolateral prefrontal cortex) of suicidal schizophrenic patients compared with non-suicidal patients. Cortical thinning was also observed in the bilateral **caudal middle frontal gyrus**, the lateral **orbitofrontal and superior frontal gyrus**, the left **pars orbitalis**, the left **pars opercularis** and the **triangularis** when comparing healthy subjects with suicidal SCZ patients. A previous study by Giakoumatos et al. (46) had found significantly less gray matter volume in **superior frontal** and **rostral middle frontal regions** in attempters compared to both non-attempers and healthy controls, and Aguilar et al. (48) reported a significant gray matter density reduction in **left orbitofrontal cortex** in patients with a history of attempt, compared with non-suicidal patients. Moreover, Rüsch et al. (49) found significantly increased white matter volumes bilaterally near the **posterior orbital** and the **inferior frontal gyri** in patients with a history of suicide attempt, compared with non-suicidal patients. Also, current self-aggression was positively correlated with white matter volume in the same regions among schizophrenic patients. The link between the **frontal region** and SB of schizophrenic patients was one of the most consistent observations across SCZ studies. Authors hypothesize that since this area is part of a cortical network known to be involved in neural processing, functions such as cognitive control of emotions, impulse control, and inhibition of inappropriate responses its dysregulation could mediate SB (45, 46, 49).

Differences in the **temporal lobe** were reported by three different articles. Besteher et al. (45) reported that SCZ patients with SB showed pronounced cortical thinning in the **right superior** and **middle temporal cortex**, compared to non-suicidal patients. Previously, Giakoumatos et al. (46) had shown that, compared to non-attempters, attempters had significantly less gray matter volumes in the **bilateral inferior temporal** and **superior temporal cortices**, and that both attempters and non-attempters, when compared to HC, had significantly decreased volumes in these regions. In addition, Aguilar et al. (48) encountered a significant gray matter density reduction in the **left superior temporal lobe** in patients who had attempted suicide when comparing with non-suicidal patients. These regions are part of the complex neuronal network that mediates the cognitive control of emotion and impulsivity. Further, as mentioned by Aguilar et al. (48), the left superior temporal lobe is known to be associated with the presence and severity of auditory hallucinations, which could result in emotional dysfunction.

Besteher et al. (45) reported significant cortical thinning in the **right superior** and **middle insular cortex** in suicidal SCZ patients compared to non-suicidal individuals. Moreover, Giakoumatos et al. (46) showed that schizophrenic suicide attempters had significantly lower gray matter volume in the **right insula** compared to both healthy controls and non-attempters. Besteher et al. (45) argue that the **insula** is implicated in delineating the boundary between self and non-self-stimuli, which is impaired in schizophrenic patients in relation to hallucinations, and that this might contribute the presence of suicidal ideation. Finally, Besteher et al. (45) detected significantly lower gray matter volume in the **left superior parietal lobe** in the schizophrenic suicide attempters compared to non-attempters. Similarly, Giakoumatos et al. (46) also reported that attempters had significantly lower gray matter volume in **supramarginal regions**, compared to non-attempters.

Additionally, a number of other brain regions were reported by only one article each. Besteher et al. (45) detected significant cortical thinning in the **right caudal anterior cingulate cortex** when comparing suicidal schizophrenic patients with healthy subjects and in the **temporopolar cortex** relative to non-suicidal patients. Giakoumatos et al. (46) mentioned that among attempters, a history of high lethality attempts in SCZ patients was associated with significantly smaller volumes in the **right cuneus**, **the left lingual gyrus**, **the bilateral pericalcarine** and right **lateral occipital area**, compared to low lethality attempters.

#### Subcortical regions

In terms of association with subcortical brain regions, Spoletini et al. (47) reported an increased volume in the **right amygdala** in patients with a history of suicidality compared to both patients without a history and HC. Giakoumatos et al. (46) described significantly lower gray matter volume in the **thalamus** of attempters, compared to non-attempters, especially when comparing low lethality attempters against non-attempters and HC. Notably, we identified no consistently SB-associated subcortical region across the five studies related to SCZ included.

### Borderline personality disorder

BPD is a psychiatric illness characterized by frenetic efforts to avoid real or imagined abandonment, unstable personal relationships that go from idealization to devaluation and persistent suicidal or self-harming behaviors. The prevalence of this condition is estimated between 0.5 and 5.9% in the general population (69). It is a disease for which few genetic studies have been performed (70), and an almost negligible amount of research has been done to determine the brain regions involved in SB and NSSI in this specific disorder. Our methodology found three neuroimaging studies investigating brain structure and suicidality in BPD patients (summarized in Table 5). Sample sizes were 26, 51, and 120 individuals from both sexes, aged 13–45 from the USA. BPD diagnoses were determined using the Diagnostic Interview for Borderlines.

#### Cortical regions

Most brain phenotypes that exhibited an association with suicidal symptoms were reported in two studies both by Soloff et al. (50, 51), with the exception of diminished gray matter volumes in the **anterior cingulate cortex** (ACC), which was associated with a higher degree of lethality in attempters by only one study (51). Consistently, Goodman et al. found a volumetric reduction in **Brodmann area 24**, which is located in the **ACC** (52). The other brain regions were effectively shared between two studies (50, 51). In 2012, Soloff et al. (51) reported that high lethality BPD attempters had significantly lower **left fusiform gyrus** volume, compared to low lethality BPD attempters. Then, in 2014, they replicated their findings. The fusiform gyrus is primarily associated with facial recognition, and it is hypothesized that a deficit of this function may affect social interactions (50).

Soloff et al. (51) found changes in the **insular cortex**, which had a lower gray matter volume in the right hemisphere of high lethality attempters. In 2014, they also described diminished gray matter volumes in the **right insula** of higher lethality attempters, compared to lower lethality attempters (50). Alterations in the insular cortex may lead patients to misjudge others' intentions and trigger disinhibited responses to perceived rejection; as this region is involved in recognition of one's own internal emotional state, perceived emotions in others, and representation of negative emotional states (50).

These studies also described a significant decrease in gray matter volume in the **left lingual gyrus** and the **right middle superior temporal gyrus** of high lethality attempters, compared to low lethality ones (50, 51). Alterations in these regions may influence social interaction due to their role in facial processing, analysis of others' intentions and reflexive responses to visual social inputs. Lastly, the **right middle inferior orbitofrontal** gyrus and cortex also showed a decrease in gray matter volume in high lethality attempters, compared to low lethality ones. The **orbitofrontal cortex** is associated with executive cognitive functions such as response inhibition, selective attention, conflict resolution, and monitoring and regulating the limbic system. Therefore, damage to this region could result in disinhibition and impulsive and aggressive behavior (50).

#### Subcortical regions

A significantly lower gray matter volume in the **left hippocampus** and **parahippocampal gyrus** of BPD high lethality attempters was observed when compared to the low lethality group (50, 51). The parahippocampal gyrus plays a role in memory encoding and retrieval, especially in the familiarity of memory scenes. It also plays a role in identifying sarcasm in verbal communication and participates in complex facial processing. An abnormality in this brain region could impair social functioning and explain SB (50, 51).

### Other disorders

Our methodology identified two further studies assessing neurobiological correlates of SB in other disorders. Their results (Table 6) included no significant differences in gray or white matter volumes between suicidal and non-suicidal patients with Panic Disorder (53) and increased pituitary volumes in Posttraumatic stress disorder (PTSD) subjects with a history of suicidal ideation compared to healthy controls (54).

### Risk of bias assessment

Most instruments for assessing risk of bias in systematic reviews are intended to be used for interventional studies, and a standard instrument for assessing neuroimaging case-control studies is yet to be established. We developed a risk of bias assessment table, partly based on the Cochrane format and in the STROBE checklist, assessing possible design and statistical biases (see Supplementary Material and methodology). Our instrument consisted of 15 items assessing potential sources of bias. This approach showed an average interrater reliability of ~68%. Notably, we detected no immediate relationship between risk of bias and interrater reliability (Supplementary Figure 1). Risk of bias across studies and constructs is reported in the Supplementary Material.

**Figure 3:**
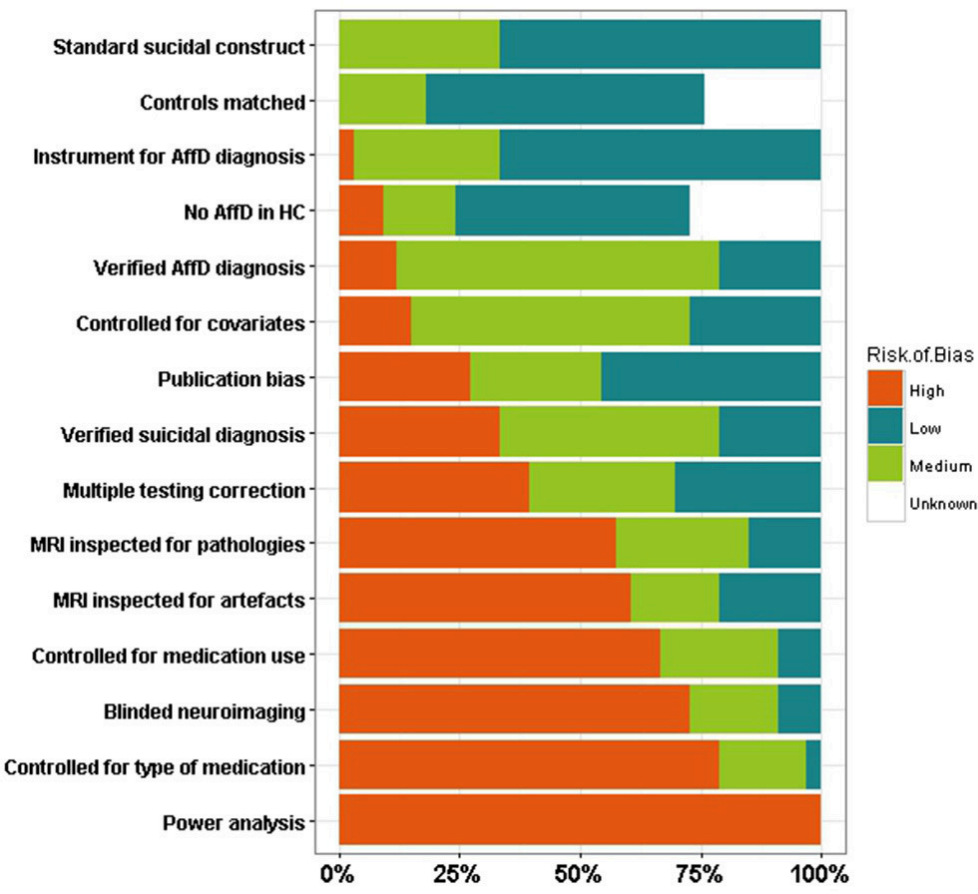
Risk of bias assessment. Stacked bars representing the percentage of literature papers identified at a high, medium, low, or unknown risk of bias for specific possible sources of bias. AffD, affective disorder; MRI, magnetic resonance imaging; HC, healthy controls.

A single construct with a high risk of bias across all studies was “***power analysis*** ***performed***” (Figure 3). The lack of inclusion of this sort of analyses could have serious consequences when interpreting non-significant results [see for e.g., (21)], as a lack of statistical evidence never implies acceptance of a null hypothesis. Other constructs with a high risk of bias across most studies included controlling for medication treatment (e.g., antidepressants) and controlling for the type of medications (Figure 3). Effects of medication on cortical phenotypes have been reported (71, 72), so controlling for this covariate is imperative to reduce the risk of bias. Constructs with a medium to high risk of bias across studies were the inclusion of covariates in statistical analyses, correcting for multiple testing and the inspection analysis for MRI artifacts and pathologies. The extent to which the possible sources of bias identified affect each study is not clear. Surprisingly, around 25% of the studies reviewed had either medium or high risk for the construct “***No mental disorder in HC***” suggesting that some studies did not assess mental health in their HC cohorts, or failed to explicitly state it in their publications, reducing their credibility. Finally, We identified that around 60% of the reviewed studies were at medium to high risk for publication bias, reporting only summary statistics and comparisons of statistical significance while not including all other comparisons performed, thus limiting our ability to compare results across studies (Figure 3).

## Discussion

Here we systematically reviewed the academic literature of neuroimaging studies investigating SB and NSSI in patients with psychiatric disorders. We found 17 articles focusing on MDD, 6 on BIP, 5 on SCZ, 3 on BPD, and single studies focusing on panic disorder and PTSD. Considering the tremendous social and economic costs that result from self-injurious behaviors, and the fact that psychiatric patients are at increased risk, it is evident that more research is needed in this area. In particular, we were unable to identify any neuroimaging studies on NSSI in psychiatric patients. Below, we discuss the most prominent consistencies and inconsistencies within and across disorders.

### Major depressive disorder

Four studies (32, 34, 35, 62) showed that white matter hyperintensities were significantly associated with a higher prevalence of past suicide attempts. These kind of lesions have also been reportedly associated with aging and dementia (73, 74), and it is unclear whether these hyper-intensities precede or are a consequence of suicidal attempts (75). The fact that these lesions were present even in samples of young adults, where they typically have a lower prevalence (74), suggests that this association might indeed be meaningful. Further, considering that associations between WMH and SB have been observed also in patients with other psychiatric disorders such as BIP, future studies should investigate whether this feature is shared among patients with suicidal symptoms across multiple disorders, and whether hyper-intensities are present in specific brain regions that could indicate which cognitive processes are affected, assuming a causal relationship which has not yet been established.

Gosnell et al. (23) and Colle et al. (24), found significantly reduced right hippocampal volumes in depressed suicidal patients. Although partially agreeing, the 2016 study (23) failed to replicate the total hippocampal volume reduction previously reported by Colle and colleagues. While comparable in several aspects, these studies used different instruments to assess suicidality, which could partially explain the differences reported. A recent ENIGMA-MDD meta-analysis with around 9,000 samples (1,700 cases and 7,200 controls) found hippocampal volume reductions present in MDD patients compared to healthy controls (76). Because of the confounding with the underlying diagnosis, it is currently not possible to establish whether hippocampal volume differences exist between patients with and without suicidality (20).

Three studies (25, 26, 31) detected reduced cortical thickness in frontoparietal regions and the insula in the left hemisphere. The concordance between these studies is surprising given the age differences (around 30, 30 and 65, respectively). However, three other studies (23, 28, 33) failed to replicate these findings. While the sample sizes of the studies ranged from 34 to 160, the number of individuals with *high suicide risk* in all studies was relatively low (between 7 and 27). Also, the study by Taylor et al. (25) only assessed suicidal ideation, or “thoughts of death,” whereas Wagner et al. (28) focused on suicide attempt. These and other methodological differences could explain the controversies, but only analyses with harmonized inclusion criteria and well-powered samples can confirm or deny the existence of these alterations.

### Bipolar disorder

Johnston et al. (39) showed that, compared with non-attempters, BIP suicide attempters had significantly lower gray matter volume bilaterally in the cerebellum and vermis. However, Baldaçara et al. (42) had previously found no volumetric differences in any of these regions when comparing attempters with non-attempters. Whilst both studies measured different variables (i.e., gray matter only vs. total volume), these results are not easily conciliated. Both studies reached different conclusions about the association between suicidal attempt and cerebellum phenotypes under BIP disorder. Comprehensive studies, considering both types of volumetric measurement and with larger samples could shed light on this current controversy. Neurobiological alterations could be used as novel suicide risk predictors for bipolar patients, but this would require studies to be replicable. We found no structures consistently associated with SB in BIP cases across the reviewed literature; this could be due to demographic, measurement or diagnosis differences across studies.

### Schizophrenia

Three out of the five studies focusing on SCZ detected associations between SB and temporal lobe volume reductions (45, 46, 48). Interestingly, the two other studies (47, 49) studied Italian populations, shared methodological approaches and came from the same groups. Therefore, they are likely to share methodological differences that explain their inconsistency with other reviewed articles. Associations between temporal lobe reductions and SB were also common in other disorders, making this an interesting candidate region. Notably, the temporal lobe is to a great extent associated with epilepsy which is also associated with suicide. An increase in suicide mortality ratio has been reported for epileptic subjects after temporal lobectomy (77), an observation consistent with the aforementioned associations.

### Borderline personality disorder

When analyzing BPD attempters, Soloff et al. both in 2012 (50) and 2014 (51) observed an association between high lethality attempters and a decrease in gray matter volume in several regions when compared to low lethality attempters. The fact that these studies tried to further stratify SB groups into high lethality and low lethality, is an example of methodological variance that has to be interpreted and harmonized in order to be comparable with results from other studies. Because these studies shared the same diagnostics, imaging, and a similar statistical methodology, and could even have a sample overlap (both recruiting as part of the outpatient program of the Western Psychiatric Institute and Clinic in Pittsburgh), the consistency of their results is not surprising. Nevertheless, we cannot rule out the possibility of some of these consistencies of being artifacts caused by a common bias. Ideally, additional evidence from independent cohorts will be needed to confirm these observations.

### Cross-disorder regions

Although SB is common in several psychiatric disorders, few efforts have attempted to map its shared neural correlates across different disorders. We hypothesized that there would be some similarities given recent reports of widespread genetic overlap across mental health disorders (22). However, the measurement of several types of cortical phenotypes (e.g., volume, surface, thickness, etc.) and suicidality constructs (e.g., ideation, attempt, severity) in the literature limited our ability to compare studies with different methodological approaches. Below we discuss the brain regions consistently associated with SB across the psychiatric disorders reviewed here.

Cortical phenotypes associated with suicidality were predominantly volume reductions or cortical thinning (Table 7). In particular, cortical thickness reduction or lower gray matter volume in the temporal cortex of suicidal patients was reported in MDD, SCZ, and BPD. As discussed above, the temporal lobe has also been associated with increased risk of suicide in epileptic subjects (77). Other cortical areas displaying reductions across disorders are the frontal, limbic, orbitofrontal, and insular lobes. Nonetheless, only reductions in both orbitofrontal and temporal cortex were recurrently reported as associated with suicidality across all four disorders reviewed.

Increases in amygdalar volume were associated with suicidality in MDD (33) and SCZ (47), and a decrease in hippocampal volume was observed in MDD (23, 24), BIP (64), and BPD (50). As previously mentioned, a reduction of this region's volume has been observed in MDD patients compared to HC (76). Furthermore, reductions in the number of synapses, arborisation, dendritic spines, and glial cell numbers have been observed in the hippocampus of depressed patients (78). Whether these changes account for the volumetric difference and are causal or consequential of MDD, as well as its association with suicidal symptoms, still remains debatable. Finally, a cerebellar gray matter reduction was consistently associated with suicidal symptoms in MDD (31) and BIP (39), although a third study in BIP reported no effect (42) (see Table 8). Interestingly, we identified no single subcortical brain region associated with suicidality across all four disorders, indicating that better powered (with bigger sample sizes and better-defined groups) studies are needed to detect associations of small effect.

**Table 8:** Subcortical phenotypes associated with SB across disorders.

	**Amygdala**	**Amygdala-hippocampus**	**Caudate nucleus**	**Cerebellum**	**Corpus Callosum**	**Hippocampus**	**Lentiform nucleus**	**Para-hippocampal region**	**Putamen**	**Midbrain**	**Thalamus**
MDD Monkul et al. (33)	Increase GM										
MDD Wagner et al. (28)	No difference	Decrease GM	Decrease GM								
MDD Hwang et al. (31)				Decrease GM			Decrease GM			Decrease GM WM	
MDD Cyprien et al. (29)					Decrease CV						
MDD Gosnell et al. (23)	No difference					Decrease CV					
MDD Colle et al. (24)						Decrease CV					
MDD Dombrovski et al. (27)									Decrease GM		
MDD Lee et al. (37)				Decrease GM							
BIP Johnston et al. (39)	No difference			Decrease GM		Decrease GM					
BIP Baldacara et al. (42)				No difference							
SCZ Spoletini et al. (47)	Increase CV										
SCZ Giakoumatos et al. (46)											Decrease GM
BPD Soloff et al. (51)						Decrease left GM		Decrease GM			
BPD Soloff et al. (50)	No difference							Decrease GM			

*CT, cortical thickness; CV, cortical volume, GM, gray matter volume; WM, white matter volume*.

## Limitations

A persistent complication we faced during the elaboration of this review was to establish whether a particular study had analyzed MRI phenotypes different from those reported in their results. In fact, by searching for non-significant or negative results throughout the studies, we detected a publication bias toward the inclusion of positive results. A significant fraction of the studies reported only statistically significant results, without including even as Supplementary Material, results for all other regions included in the study and their results (see Figure 3). Therefore, our ability to compare studies and identify potential consistencies and inconsistencies was limited by the available information. We encourage the research community to include all the results derived from their analyses, as they might be useful for informing the design of new studies and for conducting meta-analyses, which are scarce in this area (21, 55).

A possible source of bias of the present systematic review is the fact that no mean sample age exclusion criterion was used. Consequently, age-dependent effects may affect the results. We have identified no common regions consistently reported as associated with suicidal symptoms across studies with mean sample age of 60 or greater. Thus, we cannot currently conclude that neural correlates associated with suicidality differ in elderly cohorts. A detailed analysis of possible age effects on SB would be valuable, albeit outside the scope of this review.

Results of several studies reviewed here should be compared with caution, as some of them were conducted in different populations, have samples with differing sex composition (e.g., some including only males while others included only females), and encompass a wide range of ages. Although partly a limitation, as results could be specific to the samples studied, this would increase a bias toward the null, making it harder to identify commonalities across studies reviewed. In spite of this, we have observed some commonalities across studies, which is consistent with recent observations of high genetic correlation between mental health disorders (22). Furthermore, almost all studies assessed suicidal symptoms or suicide risk as part of a standard instrument used for assessing other mental disorders. The convergent and discriminant validity of these different suicide assessment instruments is not clear and makes their comparison difficult. This limiting factor is important, especially considering that a number of current instruments have been reported inadequate for SB risk assessment (13).

Finally, the lack of a standard to assess risk of bias, specifically for neuroimaging case-control studies, motivated us to generate our own. We attempted to follow Cochrane's structure while covering common sources of bias that this kind of studies might have; we partially based our analysis on the STROBE checklist (79). This is an important first step toward reproducible research in neuroimaging studies of suicidality. A key potential source of bias which was not directly addressed by our instrument is the statistical power of the studies taking into account their sample sizes. The median sample size for all studies was 66 including both cases and controls, a value well beyond the estimated sample size of ~2,000 that would be needed to detect relatively low effect sizes at whole brain study-wide significance (21). A meta-analysis of all the selected literature would be a valid approach to achieve a sufficiently powered sample and reliable results, but due to the publication bias mentioned above and the methodological inconsistencies across different studies, this approach was not feasible.

## Conclusions and perspectives

In this review we aimed to collate the results from a variety of structural MRI studies regarding specific cortical and subcortical brain regions implicated in SB and NSSI in patients with a psychiatric disorder. For all the possible combinations of selected keywords (see section Methods) we were only able to recover 50 papers from which just 6 were mainly focused on NSSI or suicidal ideation alone. Unfortunately, after selecting papers that met minimum quality standards and selecting only those that used structural MRI, we were left with 33 papers focusing on SB and no study related to NSSI. Regions most likely associated with suicidality across mental illness include the frontal and temporal cortical regions, as they were consistently reported across the disorders reviewed; as well as the hippocampus, which was implicated by four studies across three disorders.

Notably, we observed that only 11 out of 33 studies included more than 100 individuals in total, and only two meta-analyses have been published so far (21, 55). Meta-analyses and mega-analyses are powerful ways to increase the sample size and achieve better-powered analyses, and we believe the field would greatly benefit from the implementation of these approaches. The majority of the studies we identified were focused mainly on MDD, with fewer studies investigating BIP, SCZ, and BPD. Surprisingly, we found no studies relating SB with eating or anxiety disorders. Research across the variety of psychiatric illnesses might help clarify the question of whether the neural circuits involved in suicidality are shared or unique across distinct mental disorders. Finally, it is critical to standardize the technical and analytical methodologies applied to neuroimaging studies in this area. This would lead to comparable and reproducible research results, which is fundamental to shed light into the underlying mechanisms of SB and NSSI in psychiatric disease. In this regard, the recent establishment of a working group within the Enhancing Neuro-Imaging Genetics through Meta-Analysis (ENIGMA) consortium (80) is of great importance, as it will enable collaborative neuroimaging analyses of unprecedented scale.

## Author contributions

CD-B, LG-M, and MR conceived and planned the study. CD-B, LG-M, AC-G, and MR carried out the analysis, drafted, and reviewed the manuscript. All authors provided critical feedback and helped shape the research, analysis, and manuscript.

### Conflict of interest statement

The authors declare that the research was conducted in the absence of any commercial or financial relationships that could be construed as a potential conflict of interest.
