# Supplementary material for: Neuroimaging Studies of Suicidal Behavior and Non-suicidal Self-Injury in Psychiatric Patients: A Systematic Review
Source: Front Psychiatry. 2018 Oct 16;9:500. doi: 10.3389/fpsyt.2018.00500 (PMC6198177; doi:10.3389/fpsyt.2018.00500)
Supplement: Supplementary file 1 [file Data_Sheet_1.docx]

**Supplementary material for Neuroimaging studies of suicidal behaviour and non-suicidal self-injury in psychiatric patients: A systematic review.**

Authors: Carmen Domínguez-Baleón^*,1^, Luis F. Gutiérrez-Mondragón^*,1^, Adrián I. Campos-González^*,2,3^ and Miguel E. Rentería^2,3,^**^†^**

**Contents**

[Supplementary figures 1](#_Toc522866341)

[Risk of bias assessment constructs description 2](#_Toc522866342)

[Risk of bias across constructs and studies 4](#_Toc522866343)

[Study and construct codes 6](#_Toc522866344)

[Prisma checklist 7](#_Toc522866345)

[Full search strategy 10](#_Toc522866346)

[Supplementary references 11](#_Toc522866347)

# Supplementary figures


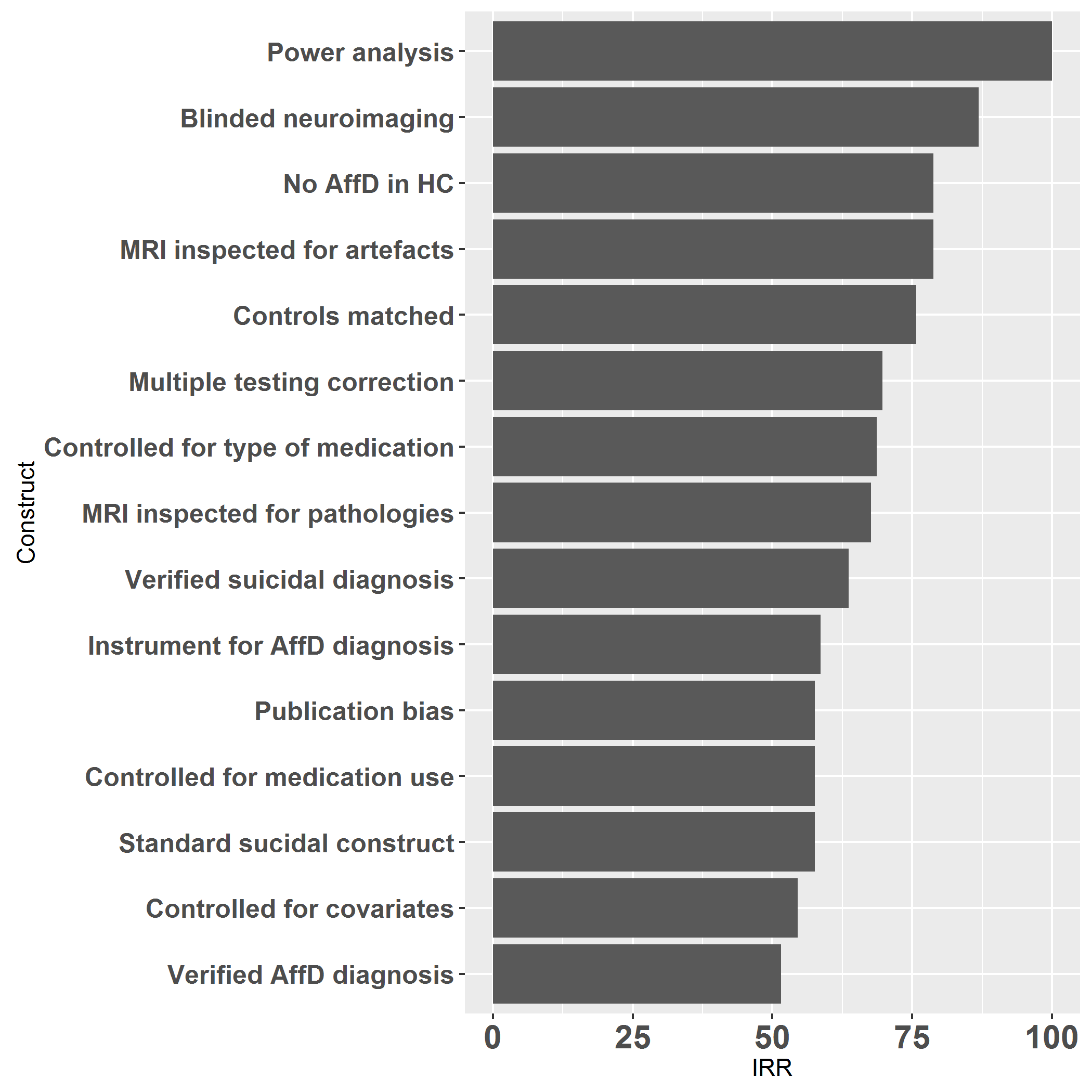


**Supplementary Figure 1 Item wise interrater reliability**

Bar plots depicting mean interrater reliability for each risk assessment construct calculated as the mean percentage agreement among raters using the *irr* R package(1).

# Risk of bias assessment constructs description

A description of potential source of bias constructs, their meaning and interpretation follows:

- **Standard suicidal construct** – This construct refers to assessing standard suicidal symptoms such as suicidal ideation or suicidal attempt. The usage of non-standard measures could range from medium to high risk if non-standard, unclear or unrelated suicidal items used.
- **Controls matched** – Whether controls, patients and suicidal patients were matched in demographics. If there were significantly different variables (non-related to case definition) then a medium risk was reported. Unknown refers to no information on the paper, notably when no controls were used (e.g. a study assessing MDD vs MDDSB) the matching was assessed between the groups studied.
- **Instruments for AD diagnosis** – Whether standard instruments (e.g. DSM-IV) were used for assessing affective disorder on the patients.
- **Controlled for covariates –** Regardless of matching, new statistical methods allow correcting for covariates and should be preferred. A study including all covariates is at low risk, while including only some or none confer a medium to high risk respectively.
- **No AD in HC** – The use of at least one standard method to ascertain healthy controls to be free of affective disorders. Unknown in this category could be due to lack of healthy controls (as described above) or lack of information in the article.
- **Publication bias** – Whether all reported regions of interest summary statistics are included in the main text or supplementary information. Hypothesis free approaches were assumed unless explicitly stating ROI in the main text.
- **Verified suicidal diagnosis** – Whether a secondary approach was used to validate the suicidality of cases and controls.
- **Blinded neuroimaging** – Whether the person in charge of performing the measurements was blinded to the status (case or control) of the subject.
- **MRI inspected for artefacts** – A study should explicitly mention whether the MRIs were subject to a blinded expert examination for artefacts.
- **Multiple testing correction** – Whether study-wise false discovery rate or family wise error corrections were performed
- **Verified AD diagnosis** – A blinded secondary independent assessment of affective disorder diagnosis reduces the probability of a false classification. A non-blinded or non-independent secondary evaluation or a lack of it would be classified as medium to high risk.
- **MRI inspected for pathologies** – A study should explicitly mention whether the MRIs were subject to a blinded expert examination for pathological findings.
- **Controlled for medication use** – Medication can have an impact on cortical thickness or volume (see main text). It is therefore crucial to at least include a dummy variable controlling for medication use.
- **Controlled for type of medication** – Because different medications could have different impacts on brain structures, whether a study should account for these using dummy variables. Using equivalents or not controlling would confer a medium to high risk of bias.
- **Power analysis** – Groups should give at least a paragraph detailing the power of their study. This helps the reader understand the degree of advance and credibility the results have, especially for negative or null results.

These guidelines were developed partially based on Cochrane(2) and the STROBE checklist, and were used by each author assessing the literature to give a low, medium or high risk score to the study. The scores were coded internally as 0, 1 and 2 and were averaged to generate the final score. Our method showed an overall moderate-high interrater reliability (~68.5%), with overall moderate to substantial mean percent agreements (50%-100%). The data were averaged across reviewers and the following cut-offs were used: a) low score X < 2/3, b) medium score 2/3<=X>=4/3, c) high score X>4/3 to summarize the data for the corresponding figure.

# Risk of bias across constructs and studies

|  | C1 | C2 | C3 | C4 | C5 | C6 | C7 | C8 | C9 | C10 | C11 | C12 | C13 | C14 | C15 |
| --- | --- | --- | --- | --- | --- | --- | --- | --- | --- | --- | --- | --- | --- | --- | --- |
| S1 | 0.00 | 0.33 | 0.00 | NAN | 1.00 | 0.00 | 1.00 | 0.33 | 2.00 | 0.67 | 1.67 | 2.00 | 0.00 | 2.00 | 0.00 |
| S2 | 0.33 | 0.00 | 0.00 | 0.67 | 0.00 | 1.00 | 2.00 | 0.67 | 2.00 | 1.67 | 1.67 | 0.00 | 2.00 | 2.00 | 0.00 |
| S3 | 0.00 | 1.00 | 0.67 | 2.00 | 1.00 | 2.00 | 2.00 | 0.33 | 2.00 | 0.33 | 0.67 | 0.67 | 2.00 | 2.00 | 1.67 |
| S4 | 0.00 | 0.33 | 0.33 | 0.00 | 0.67 | 0.00 | 2.00 | 1.33 | 2.00 | 0.33 | 0.33 | 1.33 | 1.67 | 2.00 | 1.33 |
| S5 | 0.00 | 0.33 | 1.00 | 0.00 | 1.00 | 2.00 | 2.00 | 1.67 | 2.00 | 1.67 | 2.00 | 0.33 | 0.00 | 0.00 | 1.33 |
| S6 | 0.00 | 0.00 | 1.00 | 0.00 | 1.00 | 2.00 | 2.00 | 1.00 | 2.00 | 2.00 | 2.00 | 0.00 | 0.67 | 1.00 | 1.33 |
| S7 | 0.00 | 0.67 | 0.00 | 0.00 | 0.67 | 0.67 | 0.67 | 0.33 | 2.00 | 1.33 | 2.00 | 2.00 | 1.33 | 1.33 | 0.00 |
| S8 | 0.67 | 0.00 | 0.33 | 1.00 | 0.00 | 0.67 | 2.00 | 0.00 | 2.00 | 2.00 | 2.00 | 2.00 | 1.33 | 1.00 | 1.67 |
| S9 | 0.50 | 0.33 | 0.33 | 2.00 | 0.67 | 1.33 | 0.33 | 0.67 | 2.00 | 0.33 | 1.00 | 0.00 | 1.67 | 1.33 | 0.00 |
| S10 | 0.50 | 0.67 | 0.67 | 2.00 | 0.67 | 1.00 | 0.33 | 0.00 | 2.00 | 1.67 | 2.00 | 0.00 | 1.67 | 2.00 | 0.00 |
| S11 | 1.00 | 0.00 | 0.50 | 0.00 | 1.33 | 2.00 | 0.67 | 0.67 | 2.00 | 2.00 | 2.00 | 2.00 | 2.00 | 2.00 | 0.00 |
| S12 | NAN | 0.33 | 0.33 | NAN | 0.33 | 0.33 | 1.00 | 0.33 | 2.00 | 2.00 | 2.00 | 2.00 | 2.00 | 0.67 | 0.00 |
| S13 | NAN | 1.67 | 0.33 | NAN | 0.67 | 0.33 | 1.00 | 0.00 | 2.00 | 2.00 | 2.00 | 2.00 | 1.00 | 0.00 | 0.00 |
| S14 | NAN | 0.67 | 0.67 | NAN | 1.67 | 2.00 | 0.33 | 1.67 | 2.00 | 2.00 | 2.00 | 2.00 | 1.33 | 1.33 | 0.33 |
| S15 | 0.67 | 1.00 | 0.00 | 0.67 | 1.33 | 1.00 | 2.00 | 0.67 | 2.00 | 1.33 | 1.33 | 0.67 | 2.00 | 2.00 | 2.00 |
| S16 | 0.00 | 0.00 | 0.33 | 0.00 | 0.67 | 2.00 | 2.00 | 1.00 | 2.00 | 1.67 | 1.67 | 0.67 | 2.00 | 2.00 | 0.00 |
| S17 | 0.00 | 0.00 | 0.33 | 0.00 | 1.33 | 1.33 | 2.00 | 1.00 | 2.00 | 1.00 | 2.00 | 1.33 | 1.33 | 0.67 | 0.33 |
| S18 | 0.67 | 0.00 | 0.00 | 0.00 | 0.67 | 1.00 | 2.00 | 1.33 | 2.00 | 2.00 | 2.00 | 0.00 | 0.00 | 1.33 | 1.00 |
| S19 | 0.00 | 0.00 | 0.33 | 0.00 | 0.00 | 1.00 | 2.00 | 1.00 | 2.00 | 1.00 | 1.00 | 0.67 | 0.00 | 0.00 | 0.00 |
| S20 | NAN | 0.33 | 0.33 | NAN | 1.33 | 2.00 | 2.00 | 0.67 | 2.00 | 1.67 | 1.67 | 0.00 | 2.00 | 2.00 | 1.67 |
| S21 | 0.00 | 0.00 | 0.33 | 0.00 | 1.67 | 1.33 | 2.00 | 1.33 | 2.00 | 1.67 | 2.00 | 0.00 | 2.00 | 0.67 | 1.67 |
| S22 | 0.67 | 0.00 | 1.33 | 0.00 | 0.67 | 1.33 | 2.00 | 1.33 | 2.00 | 2.00 | 2.00 | 0.67 | 2.00 | 2.00 | 0.33 |
| S23 | 1.00 | 0.00 | 1.33 | 0.00 | 0.67 | 1.33 | 2.00 | 2.00 | 2.00 | 2.00 | 2.00 | 1.33 | 1.67 | 2.00 | 1.67 |
| S24 | 0.00 | 1.00 | 0.00 | 1.33 | 1.67 | 1.00 | 2.00 | 0.00 | 2.00 | 2.00 | 2.00 | 2.00 | 2.00 | 2.00 | 1.33 |
| S25 | 0.00 | 1.00 | 0.67 | 0.00 | 0.00 | 2.00 | 2.00 | 1.00 | 2.00 | 1.33 | 1.33 | 0.00 | 0.00 | 0.00 | 0.67 |
| S26 | NAN | 0.33 | 0.00 | NAN | 1.33 | 1.00 | 2.00 | 0.67 | 2.00 | 1.67 | 1.67 | 1.67 | 2.00 | 2.00 | 1.00 |
| S27 | 0.00 | 0.00 | 0.67 | 0.00 | 1.33 | 2.00 | 2.00 | 2.00 | 2.00 | 2.00 | 2.00 | 1.67 | 2.00 | 2.00 | 0.00 |
| S28 | NAN | 0.67 | 0.00 | NAN | 0.67 | 1.33 | 2.00 | 0.33 | 2.00 | 2.00 | 2.00 | 1.67 | 2.00 | 2.00 | 0.67 |
| S29 | NAN | 0.00 | 0.00 | NAN | 0.67 | 0.33 | 2.00 | 1.00 | 2.00 | 1.00 | 1.00 | 1.67 | 2.00 | 2.00 | 1.67 |
| S30 | NAN | 0.00 | 0.00 | NAN | 0.67 | 0.00 | 2.00 | 0.67 | 2.00 | 2.00 | 2.00 | 1.33 | 2.00 | 2.00 | 2.00 |
| S31 | 0.00 | 1.00 | 0.67 | 0.00 | 1.67 | 2.00 | 2.00 | 1.67 | 2.00 | 2.00 | 2.00 | 1.33 | 2.00 | 2.00 | 1.67 |
| S32 | 0.00 | 0.00 | 0.00 | 0.00 | 0.00 | 0.00 | 2.00 | 0.67 | 2.00 | 1.00 | 1.67 | 0.00 | 0.00 | 2.00 | 1.33 |
| S33 | 0.33 | 0.67 | 1.00 | 0.67 | 0.33 | 1.67 | 1.00 | 0.67 | 2.00 | 2.00 | 2.00 | 2.00 | 0.00 | 0.33 | 0.33 |

# Study and construct codes

| Study | DOI |
| --- | --- |
| S1 | 10.1016/j.jpsychires.2014.12.010 |
| S2 | 10.1016/j.pnpbp.2015.05.001 |
| S3 | 10.1016/j.jad.2014.04.046 |
| S4 | 10.1017/S0033291711002133 |
| S5 | 10.1016/j.jpsychires.2012.07.013 |
| S6 | 10.1016/j.neuroimage.2010.08.082 |
| S7 | 10.1016/j.biopsych.2011.02.035 |
| S8 | 10.1177/0891988710363713 |
| S9 | 10.1016/j.pnpbp.2008.05.009 |
| S10 | 10.1007/s00406-007-0755-x |
| S11 | 10.1038/sj.mp.4001919 |
| S12 | 10.1097/01.chi.0000120020.48166.93 |
| S13 | 10.1016/j.jad.2005.01.007 |
| S14 | 10.1016/S0006-3223(01)01098-8 |
| S15 | 10.1176/appi.ajp.2016.15050652 |
| S16 | 10.1016/j.jad.2012.05.001 |
| S17 | 10.1016/j.neulet.2009.11.047 |
| S18 | 10.1016/j.jpsychires.2013.06.011 |
| S19 | 10.1016/j.schres.2010.08.023 |
| S20 | 10.1016/j.pnpbp.2008.06.016 |
| S21 | 10.1016/j.pscychresns.2007.12.011 |
| S22 | 10.1016/j.jpsychires.2012.01.003 |
| S23 | 10.1016/j.pscychresns.2014.02.006 |
| S24 | 10.1016/j.pscychresns.2016.09.005 |
| S25 | 10.1016/j.schres.2016.08.010 |
| S26 | 10.1111/acps.12314 |
| S27 | 10.1016/j.jad.2011.06.059 |
| S28 | 10.1016/j.jagp.2013.01.063 |
| S29 | 10.1016/j.comppsych.2016.02.013 |
| S30 | 10.1016/j.jad.2015.01.001 |
| S31 | 10.1016/j.jpsychires.2010.11.011 |
| S32 | 10.1016/j.pscychresns.2017.04.012 |
| S33 | 10.1016/j.biopsych.2003.11.021 |

| Construct | Name |
| --- | --- |
| C1 | Controls matched |
| C2 | Instruments for AD diagnosis |
| C3 | Standard suicidal construct |
| C4 | No AD in HC |
| C5 | Verified AD diagnosis |
| C6 | Verified suicidal diagnosis |
| C7 | Blinded neuroimaging |
| C8 | Case control data analysed together |
| C9 | Controlled for covariates |
| C10 | Power analysis |
| C11 | Controlled for medication use |
| C12 | Controlled for type of medication |
| C13 | Multiple testing correction |
| C14 | MRI inspected for artefacts |
| C15 | MRI inspected for pathologies |
| C16 | Publication bias |

# Prisma checklist

For this systematic review, we followed the PRISMA guidelines(3). We attach this completed checklist for review purposes, nonetheless, format differences between the resubmitted and the published manuscript may make this checklist seem inaccurate or out of sync.

| **Section/topic** | **#** | **Checklist item** | **Reported on page #** |
| --- | --- | --- | --- |
| **TITLE** | | |  |
| Title | 1 | Identify the report as a systematic review, meta-analysis, or both. | 1 |
| **ABSTRACT** | | |  |
| Structured summary | 2 | Provide a structured summary including, as applicable: background; objectives; data sources; study eligibility criteria, participants, and interventions; study appraisal and synthesis methods; results; limitations; conclusions and implications of key findings; systematic review registration number. | 2 |
| **INTRODUCTION** | | |  |
| Rationale | 3 | Describe the rationale for the review in the context of what is already known. | 4 |
| Objectives | 4 | Provide an explicit statement of questions being addressed with reference to participants, interventions, comparisons, outcomes, and study design (PICOS). | 4 |
| **METHODS** | | |  |
| Protocol and registration | 5 | Indicate if a review protocol exists, if and where it can be accessed (e.g., Web address), and, if available, provide registration information including registration number. | N/A |
| Eligibility criteria | 6 | Specify study characteristics (e.g., PICOS, length of follow-up) and report characteristics (e.g., years considered, language, publication status) used as criteria for eligibility, giving rationale. | 5 |
| Information sources | 7 | Describe all information sources (e.g., databases with dates of coverage, contact with study authors to identify additional studies) in the search and date last searched. | 5 |
| Search | 8 | Present full electronic search strategy for at least one database, including any limits used, such that it could be repeated. | 5,Table 1, & Supp. material |
| Study selection | 9 | State the process for selecting studies (i.e., screening, eligibility, included in systematic review, and, if applicable, included in the meta-analysis). | 5,Figure 1 |
| Data collection process | 10 | Describe method of data extraction from reports (e.g., piloted forms, independently, in duplicate) and any processes for obtaining and confirming data from investigators. | 5 |
| Data items | 11 | List and define all variables for which data were sought (e.g., PICOS, funding sources) and any assumptions and simplifications made. | 5 and Table 2-5 |
| Risk of bias in individual studies | 12 | Describe methods used for assessing risk of bias of individual studies (including specification of whether this was done at the study or outcome level), and how this information is to be used in any data synthesis. | 6 |
| Summary measures | 13 | State the principal summary measures (e.g., risk ratio, difference in means). | 5 |
| Synthesis of results | 14 | Describe the methods of handling data and combining results of studies, if done, including measures of consistency (e.g., I^2^) for each meta-analysis. | NA |
| Risk of bias across studies | 15 | Specify any assessment of risk of bias that may affect the cumulative evidence (e.g., publication bias, selective reporting within studies). | 6 & sup. material |
| Additional analyses | 16 | Describe methods of additional analyses (e.g., sensitivity or subgroup analyses, meta-regression), if done, indicating which were pre-specified. | N/A |
| **RESULTS** | | |  |
| Study selection | 17 | Give numbers of studies screened, assessed for eligibility, and included in the review, with reasons for exclusions at each stage, ideally with a flow diagram. | 6, Fig2 |
| Study characteristics | 18 | For each study, present characteristics for which data were extracted (e.g., study size, PICOS, follow-up period) and provide the citations. | Tables 2-5 |
| Risk of bias within studies | 19 | Present data on risk of bias of each study and, if available, any outcome level assessment (see item 12). | 16, Fig.3 |
| Results of individual studies | 20 | For all outcomes considered (benefits or harms), present, for each study: (a) simple summary data for each intervention group (b) effect estimates and confidence intervals, ideally with a forest plot. | Tables 2-5 |
| Synthesis of results | 21 | Present results of each meta-analysis done, including confidence intervals and measures of consistency. | N/A |
| Risk of bias across studies | 22 | Present results of any assessment of risk of bias across studies (see Item 15). | Fig 3. Supp. material |
| Additional analysis | 23 | Give results of additional analyses, if done (e.g., sensitivity or subgroup analyses, meta-regression [see Item 16]). | N/A |
| **DISCUSSION** | | |  |
| Summary of evidence | 24 | Summarize the main findings including the strength of evidence for each main outcome; consider their relevance to key groups (e.g., healthcare providers, users, and policy makers). | 16-19 |
| Limitations | 25 | Discuss limitations at study and outcome level (e.g., risk of bias), and at review-level (e.g., incomplete retrieval of identified research, reporting bias). | 20-21 |
| Conclusions | 26 | Provide a general interpretation of the results in the context of other evidence, and implications for future research. | 17-20 |
| **FUNDING** | | |  |
| Funding | 27 | Describe sources of funding for the systematic review and other support (e.g., supply of data); role of funders for the systematic review. | 22 |

# Full search strategy

In the present systematic review, we have queried four databases using combinations of the keywords in Table 1, and reported it in that manner for the sake of reducing manuscript length. Nonetheless, following the PRISMA guidelines, item number 8 “***Present full electronic search strategy for at least one database, including any limits used, such that it could be repeated***”, we include the full electronic search strategy for two of the databases queried which covered the majority of the literature reviewed (Fig 2).

Pubmed:

( MRI[Title/Abstract] OR magnetic-resonance imaging[Title/Abstract]) AND((((Self harm[Title/Abstract] OR Self-harm[Title/Abstract]) OR suicide[Title/Abstract]) OR suicide attempt[Title/Abstract]) OR suicidal behaviour[Title/Abstract]) AND((((((((((((((((((psychiatry[Title/Abstract] OR depression[Title/Abstract]) OR mdd[Title/Abstract]) OR schizophrenia[Title/Abstract]) OR bipolar disorder[Title/Abstract]) OR anorexia nervosa[Title/Abstract]) OR bulimia nervosa[Title/Abstract]) OR post-traumatic stress disorder[Title/Abstract]) OR ptsd[Title/Abstract]) OR alcohol use disorder[Title/Abstract]) OR cannabis[Title/Abstract]) OR alcoholism[Title/Abstract]) OR borderline personality disorder[Title/Abstract]) OR anxiety[Title/Abstract]) OR attention deficit hyperactivity disorder[Title/Abstract]) OR ADHD[Title/Abstract]) OR alcohol abuse[Title/Abstract]) OR anxiety disorders[Title/Abstract]) OR eating disorders[Title/Abstract])

Embase:

("MRI" OR "magnetic-resonance imaging" OR "magnetic resonance imaging") AND ("Self-harm" OR "Self harm" OR "suicide attempt" OR "self-injury" OR "self injury" OR "self-poisoning" OR "self poisoning" OR "self-mutilation" OR "self mutilation") AND ("psychiatry" OR "depression" OR "mdd" OR "schizophrenia" OR "bipolar disorder" OR "anorexia nervosa" OR "bulimia nervosa" OR "post-traumatic stress disorder" OR "post traumatic stress disorder" OR "ptsd" OR "alcohol use disorder" OR "cannabis" OR "alcoholism" OR "borderline personality disorder" OR "anxiety" OR "attention deficit hyperactivity disorder" OR "ADHD" OR "alcohol abuse" OR "anxiety disorders" OR "eating disorders")

# Supplementary references

1. Gamer M, Lemon J, Gamer MM. Package ‘irr’.

2. Editors PM. Observational studies: getting clear about transparency. PLoS medicine. 2014;11(8):e1001711.

3. Moher D, Liberati A, Tetzlaff J, Altman DG. Preferred reporting items for systematic reviews and meta-analyses: the PRISMA statement. Annals of internal medicine. 2009;151(4):264-9.
